# The skin‐brain connection and pleasant touch as supportive care for psychocutaneous disorders

**DOI:** 10.1002/ski2.310

**Published:** 2023-12-01

**Authors:** Bárbara Roque Ferreira, Claudia C. Aguirre, Nathalie Rapoport‐Hubschman, Abiodun O. Adewuya, Ludivine Canchy, David Morizet, Francesca Vincenzi, Francis P. McGlone

**Affiliations:** ^1^ Department of Dermatology Centre Hospitalier de Mouscron Hainaut Belgium; ^2^ University of Brest Laboratoire interactions épithéliums‐neurones (LIEN) Brest France; ^3^ Centre for Philosophy of Science University of Lisbon Lisbon Portugal; ^4^ Doctorclaudia, Inc. Los Angeles California USA; ^5^ Mind Body Medicine Institute, Founder and Director Paris France; ^6^ Private Practice Paris France; ^7^ Lagos State University College of Medicine Ikeja Lagos Nigeria; ^8^ La Roche‐Posay Laboratoire Dermatologique Levallois‐Perret France; ^9^ L’Oréal Research & Innovation, Evaluation Intelligence Clichy France; ^10^ Institute of Psychology Health & Society Liverpool University Liverpool UK

## Abstract

Psychodermatology is a subdiscipline of dermatology at the intersection of dermatology, psychiatry, and psychology. In dermatology clinical practice, patients may present with skin disease that affects their mental health, or skin disorders induced or worsened by psychological/psychiatric problems so there is a need for specialised education of dermatologists, as well as multidisciplinary teams, to achieve better management of these patients. Understanding the interaction between the central nervous system and the skin underlying psychocutaneous disorders could help identify alternative therapies that may improve patient well‐being. The concept of pleasurable touch has received increasing attention following the discovery of C‐tactile (CT) fibres. While afferent C‐fibre stimulation is usually associated with pain, temperature, or itch, CT‐fibres are stimulated optimally by a stimulus not in the nociceptor range but by a gentle, low‐force stroking. As this affective touch may counteract unpleasurable sensations, such as pain and itch, and elicit positive feelings, the potential benefits of gentle touch and massage are interesting for dermatological, especially psychocutaneous, disorders. Here we provide an overview of the skin‐brain connection to help understand the benefits of touch and massage, as illustrated with studies on atopic dermatitis and burns, as an adjunct to dermatological treatment for improving patient well‐being and optimising treatment outcomes.

1

1 **What is already known about this topic?**

- The skin‐brain connection is well established.
- While afferent C‐fibre stimulation is usually associated with pain, temperature, or itch, C‐tactile‐fibres are stimulated optimally by a stimulus not in the nociceptor range but by a gentle, low‐force stroking, pleasant touch.

**What does this study add?**

- We review evidence on how pleasant touch (gentle affective touch or massage) can be useful as supportive care for skin conditions, particularly psychocutaneous disorders, to counteract unpleasurable sensations, such as pain and itch, and elicit positive feelings to improve patient well‐being and quality of life.

## 2 INTRODUCTION

Psychodermatology, also known as psychocutaneous medicine, is a subspeciality of dermatology at the intersection of dermatology, psychiatry, and psychology; it uses a combined approach to treat conditions by addressing the reciprocal interactions between the skin and the brain. No single universal classification for psychodermatological disorders exists at this time, but some classifications have been proposed to help healthcare professionals recognise these disorders and improve patient management.^1^ In clinical practice, psychodermatology patients generally present with skin disorders that either affect their mental well‐being, or are triggered or worsened by psychological/psychiatric problems. Additionally, some patients may exhibit a skin disorder or mental illness that is linked with medications prescribed in dermatology with psychiatric consequences or medications used in psychiatry with dermatologic consequences^2^, ^3^, ^4^:

- Patients with a primary dermatological disorder that can be triggered or worsened by psychological stress (psychophysiological disorders) and that can lead to secondary psychosocial comorbidities (e.g., atopic dermatitis [AD], psoriasis, vitiligo, acne, seborrhoeic dermatitis).
- Dermatological manifestations in patients with a primary psychiatric illness (e.g., skin picking disorder, trichotillomania, factitious disorders).
- Cancer treatment‐related dermatological side effects (e.g., alopecia, rash, pruritus and nail alterations) that adversely affect self‐esteem, mental well‐being and quality of life (QoL), psychiatric consequences of some dermatological treatments (e.g., mood disorders and systemic steroids) and skin conditions secondary to their psychotropic medication (e.g., lithium may be associated with psoriasis).^2^

The stigma of visible skin disorders can have a significant impact on self‐esteem and psychological functioning of patients and psychological stress can affect the body and aggravate skin disorders, either directly via psychoneuroimmunological pathways or via behavioural reactions, leading to vicious cycles of stress and impaired skin. A nationwide survey by dermatologists in the United Kingdom reported that up to 85% of dermatology patients may have psychological issues.^5^ However, patients with dermatologic disease and psychosocial comorbidity often have difficulty gaining access to specialists for accurate diagnoses due to lack of resources^6^ and dermatologists may lack appropriate knowledge on holistic treatment to improve patient outcomes, especially patients' mental well‐being and QoL.^7^, ^8^, ^9^

Therefore, there is a dire need to raise awareness about subjective aspects in dermatology practice to offer solutions to patients that consider both dermatological and psychological dimensions which are relevant in several skin disorders. In this review article, we provide an overview of evidence for the skin‐brain connection and focus on the neuroscience of pleasurable touch to help understand how to improve the well‐being of psychodermatology patients using the potential benefits of gentle affective touch and massage.

## 3 IMPLICATIONS FOR PATIENT CARE

The psychological aspect of psychocutaneous disorders should be considered with screening for physical and mental comorbidities and referral to qualified psychiatrists or clinical psychologists when needed.^10^ Holistic care incorporating complementary and alternative medicine with a low risk profile, for example, yoga, the use of chiropractic and osteopathic practices, meditation and massage therapy, is increasingly being used as an adjunct to conventional medicine to address biological, psychological and psychosocial factors.^11^ Although randomized controlled trials (RCT) are limited, holistic approaches may have beneficial effects on the skin disorder, pruritus and pain, as well as on anxiety, stress, and sleep disorders to improve patients' well‐being.^10^ Furthermore, treatment adherence may be improved if both the psychiatric disorder and dermatological disorder are managed in everyday dermatologic practice.^12^

## 4 THE INFLUENCE OF PSYCHOLOGICAL STATE IN DERMATOLOGICAL CONDITIONS

The mind‐body connection has been recognised for centuries and clinical observations have highlighted a close relationship between psychology and dermatology. Readily visible skin conditions can negatively impact self‐image and cause feelings of isolation, loneliness, lower self‐esteem, and lower body satisfaction. Furthermore, skin disorders are often chronic conditions which may add to the psychological distress.^13^ An observational cross‐sectional study conducted with 4994 participants from dermatology clinics across 13 European countries reported significantly higher prevalence of depression (10.1% vs. 4.3%), anxiety disorder (17.2% vs. 11.1%), and suicidal ideation (12.7% vs. 8.3%), among patients with common skin diseases compared with controls.^14^

Patients often recognise that stressful events exacerbate their skin condition, while psychological stress can affect healthy skin and aggravate skin disorders (e.g., AD, acne, psoriasis, and prurigo nodularis), either directly via psychoneuroimmunological pathways or via behavioural reactions.^15^ Stress may be a precursor in the exacerbation of psoriasis and the management of psychological stress and sleep disturbances can improve QoL in these patients.^16^ At a pathophysiological level, perceived stress may dysregulate the hypothalamic‐pituitary‐adrenal (HPA) axis, sympathetic‐adrenal‐medullary axis, peripheral nervous system, and immune system.^17^, ^18^ Psoriatic lesions, which are related to an increased production of cutaneous inflammatory mediators and neurogenic inflammation, are a visible aspect of this systemic inflammation, which is also involved in the imbalance of neurotransmitters and the development of symptoms of depression and anxiety.^19^

Also, the onset, progression and severity of AD are known to be influenced by psychological stress and severe AD, particularly with severe chronic pruritus, has been linked to increased prevalence of anxiety, insomnia and depression.^20^, ^21^ Globally, stress has a significant impact on pruritic skin diseases,^22^ as it exacerbates itch, leading to a vicious cycle of stress, itching (and scratching), and sleep disorders that affect disease severity and QoL.^20^, ^23^

Patients with AD have xerosis that is hypersensitive to itch and the associated scratching induces further itching along with skin barrier disruption that allows allergens to enter the epidermis and exacerbate inflammation.^24^ The release of various itch mediators then triggers the activation of the peripheral nervous system that plays a critical role in the itch‐scratch cycle and exacerbates AD.^24^, ^25^ Although scratching may temporarily relieve itching through activation of pain sensory nerve fibres, rubbing or stroking the skin could be a less damaging alternative since, in a mouse model, gentle skin stroking reduced itch signalling in the spinal cord through the activation of low threshold mechanosensitive VGLUT3‐lineage sensory nerves.^26^

Furthermore, the involvement of the central nervous system makes AD patients sensitive to stress. Acute stress was found to reduce the perception of experimentally induced itch in AD patients but was associated with increased spontaneous off‐site scratching behaviour.^27^, ^28^ While the skin immune system dysfunction initiates pathological changes in AD, itch cognitive distortion due to alterations in the brain neural network and stress system may contribute to the progression of AD.^29^ In healthy subjects, an activation of the somatosensory cortex and motor cortex brain regions is associated with itch and scratch behaviour, whereas the activity of the prefrontal cortex, which controls other brain regions to maintain wellbeing and cognitive processes (such as inhibition and decision‐making), is unchanged. Conversely, in AD patients under itch conditions, it has been observed a dorsolateral prefrontal cortex overactivity with no changes in activation of the somatosensory and motor cortex.^29^, ^30^ Scratching‐induced brain dopaminergic reward and habit learning systems and noradrenergenic system activation can stimulate HPA axis that may cause dorsolateral prefrontal cortex disturbance leading to the vicious spiral of itch cognitive distortion.^29^ Furthermore, it has been suggested that itch cognitive distortion from prefrontal cortex disturbance may be associated with some neuropsychiatric symptoms.^29^ Hence, therapeutic interventions to reduce stress may be beneficial for reducing pruritus and skin lesions in AD, as well as related psychological symptoms.^31^, ^32^

The role of psychosocial factors, such as psychosocial stress or negative expectations, might also be relevant in “sensitive skin”, characterised by unpleasant symptoms (e.g., stinging, burning, pain, pruritus, tightness and tingling sensations) but no objective clinical features, where changes in the cutaneous free nerve endings could play a role.^33^, ^34^

Elucidation of the pathophysiological mechanisms and the interaction between the central nervous system and the skin underlying these psychocutaneous disorders is required to help understand the interactions between mental state, dermatoses, and skin symptoms to find the most suitable holistic therapies to improve patients' well‐being and QoL.

## 5 THE SKIN‐BRAIN CONNECTION

The skin shares a common ectodermal origin with the brain, spinal cord, and peripheral nerves which helps explain the likely interactions between them and the similarity between specific skin cells and neurons. Furthermore, melanocytes also share signalling molecules, receptors and signalling pathways with cells of the nervous system.^35^, ^36^, ^37^ Additionally, although the underlying pathophysiological mechanisms are not fully understood, the skin and brain are anatomically and functionally connected (skin‐brain axis) via a complex interplay between the skin and the nervous, endocrine and immune systems, with both central (systemic) and peripheral (local) pathways playing a role to modulate the response.^15^ Activation of these pathways influences the skin's immune system, barrier function, wound healing and susceptibility to infection.^15^ Many skin disorders have a complex psychoneuroimmunological pathogenesis.^13^ The skin is both a target and source of stress mediators, forming a highly selective barrier for maintaining homoeostasis, while the immune function reacts to stress.^38^

The main functions of the skin are to form a protective barrier, maintain homoeostasis and relay sensorial information to the brain, with effective communication between them.^38^

The somatosensory function serves to relay information from the skin on the immediate environment (pressure/vibration, temperature, itch, and pain), via specialised cutaneous receptors associated with sensory neurons.^39^ The classical discriminative role of touch is to detect, discriminate and identify external stimuli (discriminative input to the brain). Sensory signals are transmitted to the brain by myelinated fibres (Aβ and Aδ) and unmyelinated nerve fibres (C‐fibres).^40^

## 6 THE NEUROSCIENCE OF AFFECTIVE TOUCH

Another dimension of the skin's somatosensory system is pleasurable touch, which has been receiving increasing attention following the discovery of a specialised class of C‐fibres called C‐tactile afferents (CT) that are tuned to light gentle stroking.^41^ While afferent C‐fibre stimulation is usually associated with pain, temperature, or itch, CTs preferred stimulus is not in the nociceptor range but specifically by a gentle, low‐force stroking of mainly non‐glabrous, hairy skin, delivered at skin temperature.^41^ CT fibres have also recently been found in glabrous skin on the hand, albeit at a lower density than in hairy skin,^42^ and a meta‐analysis of 23 publications observed that the affective stimulations on hairy skin compared to glabrous skin were perceived as equally pleasant.^43^ The sensory function of touch by CT afferents is linked to pleasure and emotions (affective input) and provides a peripheral mechanism for signalling pleasant skin‐to‐skin contact in humans.^41^, ^44^ This emotional dimension of touch, called “affective touch”, refers to any tactile processing with a hedonic and/or a motivational value and is underpinned by anatomical pathways distinguishable from discriminative touch.^45^, ^46^, ^47^ While discriminative touch mainly elicits the activation of somatosensory cortex,^41^ the main target in the brain for the processing of these CT‐mediated pleasant properties of touch is the insular cortex, which plays an important role in the processing of emotions.^48^ Affective touch reduces activation of areas related to pain processing, namely the insula and anterior cingulate cortex, and reduces electrically‐induced itch, through activation of regions involved in pleasure and reward.^49^ Furthermore, the neural basis of affective touch and pain has been reviewed and CT‐optimal touch appears to be beneficial in reducing acute pain^50^, ^51^, ^52^ and chronic pain.^53^ Gentle stroking has been shown to decrease noxious‐evoked brain activity in human infants, which is similar to that observed when adults experience pain.^50^ A reduction in pain, as assessed on a visual analog scale, was observed when CT‐optimal touch was applied and this was independent of pain stimulus intensity.^51^ Similarly, affective touch between romantic couples has an inhibitory effect on pain.^52^ Furthermore, chronic generalised pain in a patient with Parkinson's disease was reduced after 7 days of affective touch applied by the partner even though the patient perceived the touch as slightly unpleasant.^53^ Strong CT‐related pain reduction was associated with low anxiety and high calmness scores obtained by a state anxiety questionnaire.^54^

The skin has a role of communication between individuals, thereby promoting affective interpersonal touch, and affiliative behaviour.^55^ Gentle or affective touch may also reduce stress and influence short‐term social interactions and well‐being by activating oxytocinergic neurons to release oxytocin, which has anti‐stress effects that improves wellbeing and may even have positive effects on health.^56^

The affective role of touch helps explain why complementary alternative medicine is a favourable active component of many manipulative treatments for well‐being and health, especially massage and related techniques.^41^, ^57^ The subjective experience of affiliative or emotional somatic pleasure of touch is especially interesting for psychodermatology since emotionally relevant CT‐mediated touch may counteract and relieve unpleasurable sensations (e.g., pain and itch) and elicit positive feelings due to overlapping and dominant effects of CT‐fibre‐mediated touch with the C‐fibre systems.^58^ Gentle affective touch could thus provide a complementary, non‐pharmacological means of treating both the psychological aspects and the perceived physical symptoms (by relieving pain and itch) of chronic skin conditions.^58^ This growing body of evidence suggests that gentle touch, or specifically affective touch^59^ which is the preferred stimulus to activate CTs, that is, low force and velocity touch delivered at skin temperature, as well as some forms of massage could relieve unpleasurable symptoms to improve the well‐being and QoL of patients with psychodermatological disorders.

## 7 EFFECTS OF TOUCH THERAPY

High‐quality studies on gentle touch, massage and related techniques are limited. A review of 11 studies on the physiological effects of interpersonal touch on patients in critical care reported significant effects of interpersonal touch such as lowered systolic and diastolic blood pressure and respiratory rate, improved sleep and decreased pain.^60^ In critical care patients, while significant effects of interpersonal touch included reduced pain, heterogeneous results were obtained on immune cells depending on the amount of pressure, body site, duration, and timing, suggesting light and moderate pressure touch may activate different neurophysiological pathways.^60^ Massage therapy has been shown to enhance immune surveillance and Natural killer (NK) cell activity in human immunodeficiency virus (HIV)‐positive patients and decreased inflammation and T helper 2 (TH2)‐type responses.^11^, ^61^ In breast cancer patients, *effleurage* massage had a short‐term effect on NK cell activity.^62^, ^63^ In another study in breast cancer patients, massage therapy (30‐min massages 3 times a week for 5 weeks) increased levels of dopamine, serotonin, NK cells and lymphocytes.^64^ Also, the effects of touch may be mediated by the density of autonomic innervation received by the body areas involved, and repetition of sessions.^60^ In a randomized preliminary study, twice‐weekly 45‐min sessions of Swedish massage over 12 weeks effectively reduced generalised anxiety disorder with the greatest benefit observed during the first 6‐week period versus the second 6‐week period.^65^ Positive verbal enhancement and the sight of touch appear to augment activation of the orbitofrontal cortex and to increase the sense of pleasantness during affective touch. Furthermore, the emotional aspects of touch are better conveyed at certain areas, for example, the forearm compared to the skin of palms and hands.^66^ Moderate pressure or light touch massage, for example, Swedish massage, has physical and physiological benefits^67^ and adverse events are extremely rare.^68^ Conversely, there may be safety concerns with non‐professional massage therapists using forceful techniques like shiatsu, urut and Rolfing.^68^ Hence stronger massage techniques will be not covered in our review.

## 8 HEALING TOUCH, THERAPEUTIC TOUCH AND AFFECTIVE TOUCH THERAPY

Complementary therapies are increasingly used as supportive care to reduce side‐effects of cancer treatment despite a paucity of robust studies. Healing touch is described as a complementary non‐invasive bio‐field therapy developed to enhance healing, the immune system and spiritual health by balancing the energy field.^69^ RCTs demonstrated a positive impact of healing touch on QoL in adult cancer patients^70^ and reductions in pain, stress, and fatigue for paediatric cancer patients and their parents.^71^ In an RCT, healing touch (using touch without muscle stimulation and non‐touch techniques) and Swedish massage therapy both improved mood and reduced pain.^72^ Similarly, both healing touch (practitioners used light, gentle touch and/or made sweeping hand motions with their hands near the patient's body) and oncology massage (therapists applied gentle pressure and kneaded patients' muscles and joints) were associated with clinically significant pain improvement in two observational studies.^73^, ^74^ On the other hand, another study observed significant declines in anxiety scores, depression, general fatigue and emotional fatigue only in the massage therapy group and not in the healing touch group.^75^

Therapeutic touch is an intentionally directed process to modulate the flow of human energy during which the practitioner uses the hands as a focus to facilitate healing; it may include physical contact (but it is not a requirement).^76^, ^77^ A review in children found insufficient evidence to recommend therapeutic touch in children, although both massage therapy and therapeutic touch seemed to elicit similar parasympathetic effects and a relaxed physiologic state with reduced stress and anxiety and improved mood.^77^

To our knowledge, only one RCT has investigated the differences of benefits between massage therapy (light/gentle *effleurage*, *petrissage* and myofascial trigger point release) and affective touch (light and consistent pressure by placing both hands on the participant for 3 min at several locations, with no side‐to‐side hand movement) with six 30‐min sessions over 2 weeks.^78^ This study showed that in advanced cancer patients with moderate to severe pain (*n* = 380 adults), both massage and affective touch showed statistically significant immediate improvements in both pain and mood (massage was superior), but there were no between‐group differences after 2 weeks.^78^

## 9 MASSAGE THERAPY AND CLINICAL RELEVANCE IN DERMATOLOGY

To illustrate benefits on well‐being of massage and gentle touch therapy as supportive care for AD and burns, we provide a brief tabulated summary of the RCTs in Table 1.

**TABLE 1:** Summary of randomized controlled trials in massage and gentle touch therapy for atopic dermatitis (AD) and hypertrophic scars/burn injuries.

Indication	Subjects	Therapy groups	Outcome
Atopic dermatitis^79^	20 children (mean 3.8 years)	10 received standard topical therapy (medications, emollients) versus 10 with standard therapy plus a 20‐min massage daily for 1 month. The massage technique was demonstrated to the parents who performed it while applying the topical therapy. Assessments were performed pre‐ and post‐therapy sessions on day 1 and after 1 month.	The parents had lower anxiety after the massage sessions. After 1 month, the parents indicated anxiety levels of their children decreased. The children in the massage group significantly improved according to independent behavioural observations for clinical measures of erythema, scaling, lichenification, excoriation, and pruritus. The control group only significantly improved on scaling.
Atopic dermatitis^80^	66 full‐term infants ≤12 months old	Infants with eczema were randomized to routine care versus routine care and 10‐min massage by the mother once daily (evening) 6 times per week. After 2 months, assessments included growth indexes, EASI, IDQOL, SAS and SDS. Eczema relapse rate was assessed at 5 months.	Compared with routine care, the massage group showed signiﬁcantly lower scores of EASI and IDQOL (both *P* < 0.001) in infants, and signiﬁcantly lower scores of SAS and SDS in mothers (both *P* < 0.01). The massage group had a lower eczema relapse rate at 5 months (*P* < 0.01).
Burns debridement^81^	28 adults	RCT of 20‐min Swedish massage stroking once a day for 1 week plus standard treatment versus standard treatment control. Before and after therapy, assessments included STAI, behaviour observation, cortisol assays. Pain assessments (MPQ, Present pain intensity scale, VAS) and POMS (depression, anger, vigour scores) were assessed on day 1 and day 7.	Immediate effects after the massage sessions were anxiety and cortisol (stress hormone) levels decreased. After 7 days, pain ratings were lower, and anger and depression had improved in the massage group compared to the control group.
Wound healing of burns^82^	20 adults (mean age 38.2 years)	During the remodelling phase of wound healing with severe itching, patients were randomized to receive 30‐min localised massage with cocoa butter to scar tissue twice a week for 5 weeks versus standard treatment control group (cocoa butter applied without massaging). Assessments included itch rating, MPQ, STAI, and POMS.	Massage immediate effects and after 5 weeks were reduced pruritus, pain, and anxiety and improved mood.
Burns^83^	24 young children (mean age 2.5 years)	Before debridement or dressing change, children hospitalised for severe burns were randomized to receive standard dressing care versus 30‐min massages (moderate‐pressure) to body areas that were not burned in addition to standard dressing care.	Children in the massage therapy group were less distressed during the dressing change procedure. Children without massage therapy responded to the dressing change procedure with increased facial grimacing, torso movement, crying, leg movement and reaching out.
Burns^84^	63 adolescents (mean age 14 years)	Participants were assigned to 15‐min massage (moderate pressure) by a trained massage therapist and standard medical care versus standard medical care for 5 weeks	Massage therapy reduced pain, pruritus, and state anxiety between baseline and the last day of the study (*P* < 0.001).
Burns^85^	284 children (aged 5 weeks to 13 years)	RCT comparing the effect of massage versus massage with aromatherapy oil on relaxation levels and distress levels in hospitalised paediatric burn patients. Itch and pain were assessed by VAS and anxiety by the STAI.	Evaluating massage for relaxation proved difficult in young children; massage therapy with or without essential oil was not effective in reducing distress behaviour scores or heart rate.
Burns^86^	240 patients (mean age 32 years)	RCT allocated burn patients to conventional primary care (control) versus 20‐min Swedish massage by a trained researcher versus 20 min music‐plus‐massage once a day for 3 consecutive days.	Decreased pain and anxiety intensity and increased relaxation levels were observed in the intervention groups (both massage and massage‐plus‐music) when compared to the control group.
Burn injury^87^	52 patients (mean age 43 years)	The intervention group received 20 min of foot reflexology massage for 3 days versus control routine care for burn‐specific pain anxiety and sleep quality.	Foot reflexology massage reduced pain anxiety, improved sleep quality, and sleep quantity with significant differences between the two groups.
Hypertrophic scars^88^	30 paediatric outpatients (mean age 59.4 months)	Therapeutic friction massage sessions of 10 min per day in combination with pressure garments versus pressure garments alone for 3 months. Patients (if able) or their care providers were instructed in the massage procedure.	The study failed to demonstrate any appreciable effects of massage therapy on the vascularity, pliability, and height of the scars. Some patients indicated a decrease in pruritus.
Hypertrophic scar after burn^89^	146 adults	Standard rehabilitation therapy for hypertrophic scars versus standard therapy plus burn scar rehabilitation 30‐min massage therapy 3 times a week. Assessments included pain and itch VAS.	After a mean duration of 34 days of burn rehabilitation massage therapy, improvements were observed for pain, pruritus, and scar characteristics (thickness, melanin deposition, erythema, TEWL, and elasticity). The decrease in pain may be due to the stimulation of afferent nerve fibres with *effleurage*‐style massage, reducing nociceptive sensations via the central nervous system.
Hypertrophic scar after burns^90^	60 adults	Intra‐individual scars were randomized to usual care control or massage therapy plus usual care. Massage, occupational or physical therapists provided massage treatment 3 times per week for 12 weeks.	When baseline measures, the control scar, and time were incorporated in the analysis, there was no evidence of long‐term benefit of massage therapy on elasticity, erythema, and pigmentation.

Abbreviations: DS, depression scale; EASI, eczema area and severity index; IDQOL, infantile dermatitis quality of life index; MPQ, McGill pain questionnaire; POMS, profile of moods state; RCT, randomized controlled trial; SAS, self‐assessed anxiety scale; STAI, state‐trait anxiety inventory; TEWL, transepidermal water loss; VAS, visual analogue scale.

### 9.1 Atopic dermatitis

An evidence‐based guideline in 2014^91^ and a systematic review in 2020^92^ of adjunctive and complementary therapies for the management of AD and treatments for flare prevention concluded that there was insufficient evidence to make recommendations. However, in 20 children with AD receiving standard therapy (topical corticosteroids, emollients and antihistamines) or standard therapy plus a daily 20‐min massage given by their parents for 1‐month, lower (subjective) anxiety levels in the children and significant improvements from baseline in (objective) clinical measures of certain AD signs, including erythema, scaling, lichenification, excoriation, and pruritus were observed (see Table 1).^79^ No adverse effects were reported. Although no causality can be determined from this unblinded study, a possible reason for the observed improvements in the AD signs may have been that the massages reduced anxiety levels in the parents and children with positive psychological effects. Also, it has been suggested that the massage therapy might be beneficial in AD as a stress‐reducing interaction between parent and child, by increasing peripheral circulation, and by increasing compliance and adherence with topical treatments^92^ and longer‐term studies are required.^93^

A 10‐min back massage was shown to improve sleep quality, sleep duration, breathing and anxiety in intensive care unit patients.^94^ Similarly, massage may be used to improve sleep, anxiety and QoL in AD patients, the majority of whom have difficulty both falling and staying asleep due to pruritus.^95^, ^96^ Massage may improve relaxation and sleep but there are no RCTs on the effect of massage on sleep in AD patients.^96^

Another study was a small pilot study in 8 children randomized to receive counselling and massage with essential oils versus without essential oils for 8 weeks.^97^ Although the AD improved in both groups, there was no significant difference in improvement shown between the massage groups with or without essential oils and subsequent periods of treatment may suggest adverse effects of the essential oils. However, massage therapy used as a complementary therapy in conjunction with normal medical treatment, appeared to help alleviate the symptoms of AD.^97^

### 9.2 Burns and scars

Depression and post‐traumatic stress are common psychiatric comorbidities following burn injury.^98^ Massage therapy is interesting for hypertrophic scars, especially after burns, as they are painful and itchy with significant psychological impact.^99^ Pruritus is, indeed, one of the most frequently encountered symptoms following burns and is challenging to treat.^100^

Literature reviews on the effectiveness of massage in scar management have found weak evidence from various regimens, non‐standardized outcome measures and various protocols,^101^ and further studies are needed.^102^ A systematic review on massage for hypertrophic burns scarring found poor quality evidence suggesting that scar massage may improve scars, pain, pruritus and mood.^103^ Finally, recent systematic reviews found scar massage significantly improved burn scar formation and reduced pruritus and anxiety,^104^ and that massage therapy interventions significantly reduced pain and anxiety intensity in burn patients.^105^

Overall, the studies in Table 1 suggest that massage therapy and pleasant touch could be used to reduce pain and pruritus, as well as reducing psychological distress, including anxiety, anger and depression, while improving mood and relaxation. There is a lack of consensus among burn centres as to the best products to use; a moisturising cream for scar massage has been recommended, and is usually used for 10 min, 3 times a day.^106^

## 10 MASSAGE THERAPY AND CANCER

Several studies of massage for cancer patients may have clinical relevance to dermatology for the management of dermatological side effects, as also for reducing anxiety, pain, fatigue, sleep disturbance, and improving QoL. Cancer treatments are often aggressive and may be associated with many unpleasant cancer‐related symptoms (e.g., pain, chemotherapy‐induced peripheral neuropathy, intense fatigue and mood disturbances) and cancer treatment‐related dermatological side effects (e.g., pruritus, rash, erythema, alopecia, and nail alterations), which can cause physical and emotional distress and have an adverse effect on QoL. Massage therapy may be a useful, cost‐effective, non‐invasive alternative treatment to improve patient well‐being in supportive cancer care.^107^ Massage may interrupt the cycle of psychological distress by increased blood and lymphatic circulation, potentiating analgesic effects, decreasing inflammation and oedema, manual release of muscle spasms, increased endogenous endorphin release and competing sensory stimuli that override pain signals inducing a pleasant feeling and relaxation.^78^ Also, studies supported the practice of massage as a nonpharmacologic pain management strategy for cancer patients.^73^, ^74^ However, while surveys indicate that the demand from cancer patients for massage as a nonpharmacologic supportive care during treatment is increasing, massage is not always incorporated into care at many cancer centres.^108^, ^109^

Several systematic reviews have been performed on the effectiveness of massage therapy on cancer symptoms and for supportive palliative care in adults^110^, ^111^, ^112^, ^113^, ^114^, ^115^ and children.^116^ Recent reviews on the effect of massage on cancer pain recognise massage therapy as an effective intervention for immediate effect on cancer pain and may improve physical function and global well‐being, although long‐term sustained benefits are less clear.^117^, ^118^ Generally, evidence on the benefits of using massage for reducing anxiety, pain, fatigue, sleep disturbance, and improving QoL was inconclusive and more rigorously designed, large‐scale, sham‐controlled RCTs are needed. A large‐scale retrospective study in 1290 cancer patients found massage therapy (standard Swedish massage, light touch massage, or foot massage for about 20 min) was associated with around 50% improvements in pain and anxiety scores.^119^ In another retrospective study in 1764 cancer patients, massage therapy was associated with reduced depressive symptoms, albeit music therapy was associated with a greater reduction than massage therapy.^120^

As a body of evidence, the numerous small RCTs (study size ranging from 21 patients to 183 patients) of short duration in adult cancer patients, show at least short‐term, immediate effects of massage on cancer‐related symptoms of fatigue,^121^, ^122^, ^123^, ^124^ anxiety^125^, ^126^ and depression,^64^, ^127^ perceived stress levels^128^ and mood disturbance,^129^ pain,^129^, ^130^, ^131^, ^132^, ^133^, ^134^ sleep disorders^131^, ^133^, ^135^ and QoL,^134^, ^136^, ^137^ with often favourable effects observed on multiple symptoms.

Massage therapy may be even more interesting for children with cancer as a safe, non‐pharmacological approach to help relieve multiple symptoms, namely anxiety.^138^, ^139^, ^140^, ^141^ In RCTs in children with cancer, massage therapy has been suggested to reduce pain,^142^ anxiety levels,^143^ and increase night time and overall sleep.^144^ While cancer treatment‐related procedures are likely to be traumatic and painful to a child, affective and interpersonal touch from the parents/caregivers and non‐essential touch from nurses may help reduce the child's distress by making them feel safe and comforted.^145^, ^146^

## 11 FUTURE PERSPECTIVES

Dermatologists are increasingly becoming aware of the benefits of a holistic approach by combining medical treatments with nonpharmacological interventions to treat psychocutaneous disorders. Massage therapy is a simple, noninvasive, and cost‐effective approach that can be used to improve patient well‐being. However, further studies are required as good quality randomized and double‐blind trials on gentle touch and massage therapy are lacking, partly because the participants cannot be blinded to their treatment, and it is not a cure.

While emollients may in some cases be used as part of a massage regimen, there is a paucity of studies investigating touch/massage to improve the effectiveness of emollients as dermocosmetic or therapeutic products and the potential synergistic effect of gentle affective touch (and self‐massage) on dermocosmetic application. For example, future studies are warranted on the effect of emollients and touch or gentle massage for the basic treatment of xerosis in the management of AD.^147^ Also, on the use of massage therapy for preventing or reducing scars (e.g., acne, surgical, traumatic scars), especially for scarring on the face and visible regions of the body that can have both a psychological (anxiety and depression) and physical impact (pain, tenderness, pruritus) on the patient.^148^ Teaching patients to massage their scars as soon as the wound is healed with a non‐perfumed emollient cream with a gradual build‐up of pressure in small circular motions, preferably twice a day, was previously reported to help prevent scars,^148^ but mixed results have been obtained.^88^, ^103^

Massage is also useful for cancer treatment‐related dermatological side effects (e.g., pruritus, radiation dermatitis and nail alterations). In a randomized cross‐over study in patients with breast cancer, the prophylactic application of niacinamide‐containing emollient was shown to prevent chemotherapy‐induced cutaneous symptoms (e.g., pruritus, xerosis) with some positive effects on QoL.^149^ Additionally, an RCT in patients treated for non‐metastatic breast cancer showed significant improvements in cancer treatment dermatological side effects (xerosis, skin infiltration, nail toxicity) after a 3‐week hydrotherapy treatment, including emollient baths and wrapping, and massages by a physiotherapist.^150^

Finally, it could be interesting to investigate the ability of affective/interpersonal touch to relieve both subjective (e.g., pain, pruritus, anxiety) and objective symptoms (e.g., lesions) and improve well‐being and treatment outcomes, for example, in AD patients.

## 12 CONCLUSIONS

Pleasant touch (gentle affective touch or massage) appears to be a good strategy to improve some skin conditions, particularly psychophysiological skin disorders (dermatoses that are triggered or worsened by stress), such as AD, and their psychological and psychiatric consequences as the interconnectedness of skin and brain can all be impacted by touch and massage. Potential benefits of massage therapy are the improvement of anxiety symptoms, fatigue and sleep disorders, decreased physical symptoms of pain and itch, and also improved immune function and wound healing. Gentle affective touch or massage therapy could be integrated into dermatology clinical practice for several dermatoses, especially chronic disorders (e.g., AD and other aetiologies of chronic pruritus), as well as in the management of cancer treatment‐related skin symptoms, using gentle massage to distribute emollients/dermocosmetics onto the skin to improve absorption and increase compliance/adherence to treatment.

## CONFLICT OF INTEREST STATEMENT

David Morizet and Francesca Vincenzi are employees of L’Oréal and Ludivine Canchy is an employee of La Roche‐Posay Laboratoire Dermatologique (L’Oréal). Abiodun O. Adewuya has nothing to disclose. Claudia C. Aguirre has received consultant, advisor, and/or speaking fees from various skincare companies, including L’Oréal. Barbara Ferreira has been a board member for La Roche‐Posay and has been a speaker in a previous lecture related to psychodermatology organised by Novartis.

## AUTHOR CONTRIBUTIONS

**Bárbara Roque Ferreira**: Conceptualisation (equal); Writing – review & editing (equal). **Claudia C. Aguirre**: Writing – review & editing (equal). **Nathalie Rapoport‐Hubschman**: Writing – review & editing (equal). **Abiodun O. Adewuya**: Writing – review & editing (equal). **Ludivine Canchy**: Writing – original draft (lead); Writing – review & editing (equal). **David Morizet**: Writing – review & editing (equal). **Francesca Vincenzi**: Writing – review & editing (equal). **Francis P. McGlone**: Conceptualisation (equal); Writing – review & editing (equal).

## ETHICS STATEMENT

Not applicable.

## Data Availability

No new data was generated.
